# Ecology of Anti-Biofilm Agents I: Antibiotics *versus* Bacteriophages

**DOI:** 10.3390/ph8030525

**Published:** 2015-09-09

**Authors:** Stephen T. Abedon

**Affiliations:** Department of Microbiology, The Ohio State University, 1680 University Dr., Mansfield, OH 44906, USA; E-Mail: abedon.1@osu.edu; Tel.: +1-419-755-4343; Fax: +1-419-755-4327

**Keywords:** antibiotics ecology, biocontrol, biofilms, biofilm control, biofilm eradication, ecology, Lanchester’s laws, phage therapy

## Abstract

Bacteriophages, the viruses that infect bacteria, have for decades been successfully used to combat antibiotic-resistant, chronic bacterial infections, many of which are likely biofilm associated. Antibiotics as anti-biofilm agents can, by contrast, be inefficacious against even genetically sensitive targets. Such deficiencies in usefulness may result from antibiotics, as naturally occurring compounds, not serving their producers, in nature, as stand-alone disruptors of mature biofilms. Anti-biofilm effectiveness by phages, by contrast, may result from a combination of inherent abilities to concentrate lytic antibacterial activity intracellularly via bacterial infection *and* extracellularly via localized population growth. Considered here is the anti-biofilm activity of microorganisms, with a case presented for why, ecologically, bacteriophages can be more efficacious than traditional antibiotics as medically or environmentally applied biofilm-disrupting agents. Four criteria, it can be argued, generally must be met, in combination, for microorganisms to eradicate biofilms: (1) Furnishing of sufficiently effective antibacterial factors, (2) intimate interaction with biofilm bacteria over extended periods, (3) associated ability to concentrate antibacterial factors in or around targets, and, ultimately, (4) a means of physically disrupting or displacing target bacteria. In nature, lytic predators of bacteria likely can meet these criteria whereas antibiotic production, in and of itself, largely may not.

## 1. Introduction

“*Chronic infections… are very difficult, if not impossible, to cure with antibiotics*.”

—T. Bjarnsholt [1]

Biological control, or simply biocontrol, is the application of organisms or their products to environments to reduce numbers of other, undesirable organisms [2]. This can include the targeting of undesired microorganisms by other microorganisms [3]. Defined broadly [1], environments include not just naturally occurring ecosystems but also man-made or man-altered circumstances. These include as seen in the context of agriculture, or can consist instead of the bodies of organisms with their associated microbiota. A number of biocontrol agents exhibit antibacterial activities and therefore are useful towards modifying the presence or distribution of bacteria. One means by which such antimicrobial biocontrol can operate is by exerting what can be described as microbial antagonism [4].

Though not typically thought of in these terms, nonetheless antibacterial biocontrol agents can include antibiotics. This is because antibiotics, as traditionally defined, are naturally occurring molecules that have been purified from bacteria or fungi, especially bacteria and fungi that have been isolated from soil [5]. Chemically, antibiotics are relatively small molecules (<<10,000 Da). Microbes are known to generate even smaller molecules (<300 Da) [6], collectively known as volatile organic compounds (VOCs), that can also possess antimicrobial properties [7]. Included as well among naturally occurring, microorganism-produced antibacterial agents are bacteriocins, which typically display a greater specificity than antibiotics [8]. Also naturally occurring are the similarly more specific, though much larger, so-called “Tailocins” [9], a.k.a. R-type pyocins [10]. In addition are bacteriophages, the viruses that infect bacteria [11]. The application of any of these agents to environments, or their production by environmentally seeded microorganisms, may be employed to effect antibacterial biocontrol.

The targets of antibacterial agents can be differentiated into specific bacterial types. These targets can include single-species infections *versus* mixed infections or instead the collective targeting of multiple species using disinfectants. Mixed targets typically will require broader-acting antibacterial formulations than single targets, or the use of cocktails in the case of biocontrol using phages [12,13,14,15]. The breadth of activity of antibacterial agents also can be important to the treatment of planktonic bacteria *versus* biofilms since bacteria found within biofilms often are less susceptible to antibiotics [16,17]. In terms of the development of antibacterial biocontrol, it is relevant to consider whether this reduced activity against biofilms might be a consequence of the natural ecology of antibiotics. Specifically, to what extent, logically, are antibiotics—as natural and, along with antibiotic-resistance mechanisms, ancient microbial products and functions [18]—in fact utilized by their microorganism producers towards clearing naturally occurring, otherwise intact bacterial biofilms? In other words, to what degree did antibiotics, prior to their discovery and subsequent harnessing as antibacterial agents, serve as effective anti-biofilm agents? Similarly, we can consider the potential for non-antibiotic antibacterial agents, including bacteriophages, to disrupt mature biofilms in nature.

Antibiotics alone clearly can kill or at least inhibit the metabolism of sensitive bacteria. Phages as predators of bacteria, however, may serve as superior anti-biofilm agents in comparison, especially, to the use of antibiotics alone. Unlike antibiotic-producing bacteria or fungi that may inhibit target organisms as a means of gaining a competitive advantage, the killing and subsequent disruption of bacteria by phages is an integral part of the phage lifecycle and therefore crucial to their survival and propagation. This antibacterial activity, for predators of bacteria, may result in anti-biofilm activity as well. For non-predator, antibiotic-producing organisms, by contrast, antibiotic-mediated growth inhibition of microorganisms might serve to enhance producer competitive abilities, but likely is less crucial to producer survival and reproduction. Furthermore, in nature the substantial killing or otherwise removal of bacteria that are biofilm-associated may require more than what the action of individual, especially relatively small-molecule chemical compounds can on their own facilitate. Such activity nevertheless typically is explicitly what is demanded of antibiotics as antibacterial drugs. The potential for antibiotics *versus* bacteriophages to disrupt intact biofilms in nature is considered here, with emphasis primarily on the potential ecology of antibiotic action against biofilm-associated bacteria. Part II of this analysis [19] focusses on the population dynamics of phage exploitation of bacterial biofilms along with the actual practice of phage-mediated biocontrol of bacterial biofilms.

### Antibiotics and Biofilm Disruption

An unfortunate feature of antibiotics as antibacterial agents is that they do not always work, at least not as well as one might hope, e.g., [20]. This, in combination with concerns over growing resistance to antibiotics [21,22], the impact of antibiotics on non-target normal-microbiota bacteria (which can lead to short-term as well as long-term health issues [23,24]), and the problem of release of antibiotics into environments resulting in potential public health consequences [25,26,27], has prompted a search for alternative, selectively toxic antibacterial agents [28]. This includes a search for alternative antibacterials that can serve as anti-biofilm agents [29]. Biofilm bacteria in particular can display a reversible tolerance to antibiotics [30,31] and, as a consequence, tend to be less easily treated using antibiotics than planktonic bacteria [16,17].

Antibiotics, as traditionally defined, are bacteriostatic or bactericidal chemical compounds that are produced by microorganisms, most notably by various bacteria and fungi that reside in soils. Antibiotics additionally can be viewed as secondary metabolites that may or may not serve in nature primarily as antibacterial agents [18,32,33]. Notwithstanding this alternative perspective, antibiotics are selectively toxic in the sense that their actions can negatively impact a subset of bacterial types but, notably, not directly damage the antibiotic-producing organism itself. This selective toxicity, independent of whatever utility the toxicity may provide in nature, is what makes antibiotics useful to us as antibacterial agents. They can interfere with the metabolism of nuisance or pathogenic bacteria without substantially negatively impacting, for example, our own tissues. Generally the impact of antibiotics also is density dependent, with low antibiotic densities possessing lower or no antibacterial activities relative to higher concentrations.

Antibiotics are not completely lacking in toxicity towards non-target organisms, including toxicity towards our own tissues at higher antibiotic densities. Particularly such negative impact towards our own tissues occurs when *in vivo* antibiotic concentrations come to exceed what can be described as minimum toxic concentrations. This density-dependent toxicity in combination with the density dependence of antibiotic effectiveness—particularly the existence of minimum inhibitory concentrations (MICs)—serves to place limits on the clinical effectiveness of specific antibiotics against specific target bacteria. Antibiotic doses that in principle could be used to eradicate problem bacteria, or eradicate problem biofilms, therefore may not be achievable due to concerns over antibiotic toxicity. This issue occurs should an antibiotic’s MIC—or minimum biofilm inhibitory concentration (MBIC) or biofilm bactericidal concentration (BBC) [17]—exceed some measure of an antibiotic’s toxic concentration, e.g., the concept of a therapeutic index. An important additional consideration, though one not addressed here, is that bacteriophages, contrasting antibiotics or other small-molecule antibacterials, are less likely to display pharmacologically emergent properties such that, properly characterized, new phages with promising *in vitro* activities are less likely to unexpectedly display *in vivo* toxicities upon animal testing [34].

In practice, biofilms can be found in association with chronic bacterial infections generally, wound infections, chronic lung infections of cystic fibrosis patients, in association with in-dwelling devices such as catheters, or on environmental surfaces, e.g., [1,35]. Infections can be associated with bacterial pathogens that have acquired antibiotic resistance. In addition, a subset of bacteria making up a given biofilm can display persister phenotypes, providing a temporary antibiotic resistance [36,37]. The reasons that antibiotics often have less than desirable antibacterial properties against biofilm-associated bacteria thus tend to stem from multiple sources, such as selective toxicity, dependence of activity on concentration, and multiple biofilm mechanisms of antibiotic resistance or tolerance as well as problems of toxicity that can be associated with applying antibiotics at very high concentrations to patients or environments.

A possible cause of some of these limitations is considered, and this is that antibiotic production may not have evolved, in some or many cases, specifically to serve as stand-alone disrupters of intact biofilms. This could be the case because of one or more of the following, with counter arguments presented parenthetically:

(1) Biofilms at least in part tend to be inherently resistant to antibiotics, that is, selectively toxic chemical agents that are not applied at extremely high concentrations (though antibiotics such as colistin do exist which are effective at targeting less metabolically active bacteria, though in this case there is also noticeable toxicity to human tissue as well);
(2) Highly efficacious, broadly acting anti-biofilm compounds may be difficult for organisms to produce or deploy without harming themselves (see, however, the newly discovered, broadly acting anti-biofilm protein, BL-DZ1 [38]);
(3) Highly efficacious but narrowly acting anti-biofilm agents may not possess sufficient ranges of activity to justify the costs to organisms of producing them, or for us to develop them as pharmaceuticals (though narrowly acting anti-biofilm agents nonetheless do exist, such as bacteriocins); or
(4) The utility of breaking up existing, intact biofilms through the use of antibiotics alone might not be sufficiently compelling to antibiotic-producing organisms to result in the evolution of antibiotics with highly effective anti-biofilm activities (though, in fact, there are numerous suggestions that the competitiveness of biofilm-producing bacteria may be enhanced through the production of antibacterial substances).

As can be seen, none of these possibilities are conclusive, suggesting that an ecological explanation for shortcomings of antibiotics as anti-biofilm agents may not be easily drawn. Here, nonetheless, detailed examination is provided of the potential for microorganisms to employ antibiotics especially as stand-alone means of substantially disrupting bacterial biofilms. Following this analysis equivalent ecological consideration is provided of the potential for bacteriophages to do the same.

## 2. Biofilm Disruption by Microorganisms

Emphasis in this and the following section is on the question of whether antibiotic production by bacteria or fungi has logically evolved for the sake of profoundly disrupting especially mature, intact, naturally occurring bacterial biofilms. This particularly is profound biofilm disruption without the aid of additional compounds or mechanisms. Notably, such more or less stand-alone biofilm-clearing or at least bacteria-killing activity is what antibiotics often are called upon to achieve in the treatment of, for instance, biofilm-associated chronic bacterial infections. Such limitations could be because eliminating mature biofilms does not happen to be the primary biological function of antibiotics, either because of an inherent lack of utility to such disruption (points 3 and 4, above) or instead because, in practical terms, biofilm removal from surfaces using a single, stand-alone, small, selectively toxic compound simply may be difficult for antimicrobial-producing organisms to achieve (points 1 and 2, above). Implicit to these arguments is the assumption that antibiotics serve in nature as antibacterial agents, e.g., as contended by Stallings [39] and Williams *et al.* [40]. This is a perspective, however, which is questioned by some, e.g., Gottlieb [5] and Davies [32], or at least alternative functions for antibiotics have been proposed [41] or additional capabilities demonstrated [42].

### 2.1. Differentiating among Potential Utilities of Antibacterial Action

**Figure 1:**
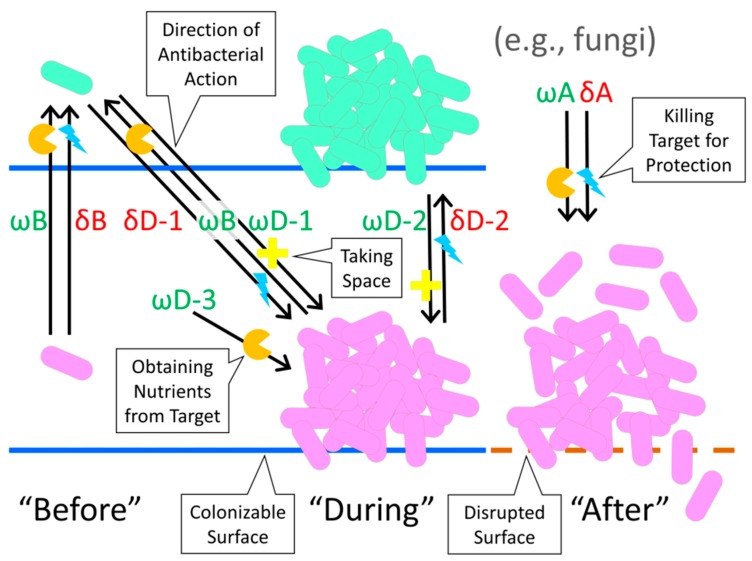
Diagrammatic representation of material presented in Table 1. Arrows point in the direction of antibacterial action, from antibacterial producer to antibacterial target. As abbreviated: “ω” refers to “Offense”, “δ” to “Defense”, “B” to “Before”, “D” to “During”, and “A” to “After”. Orange “Pacman”-like symbols refer to antibacterial deployment that is followed by consumption of target organisms for nutrients, turquoise lightning bolts refer to antibacterial-mediated killing of target organisms for the sake of protection of producing organisms, and yellow crosses refer to antibacterial action against target organisms for the sake of obtaining colonizable surfaces (*i.e.*, “Space”) by producer organisms. The solid, blue horizontal lines refer to intact colonizable surfaces while the brown dashed line refers to an equivalent but disrupted surface. The arrow labeled with ωD-3 (see also Table 1) refers to as effected by either disseminating or instead biofilm-associated bacteria. A second δB arrow, equivalent in placement to the second-from-the-left ωB arrow, has been omitted from the figure to reduce clutter. The actions indicated in the right-hand third of the figure are as potentially effected by fungi.

The biofilm “Life cycle” involves cell adherence, such as to a surface, which is followed by cell population growth and extracellular polymeric substance (EPS) production [43]. Subsequent cell dispersal can occur via a number of mechanisms, which often include the release of individual (single), now-planktonic, dispersing cells, though also can involve instead the release of clumps of dispersing cells [44]. In considering the possible utility of anti-biofilm activity to antibiotic-producing organisms, target bacteria can be distinguished in terms of this life cycle. Categories of bacterial targets thereby may include (1) those that are minimally clumped and/or still planktonic prior especially to surface colonization (referred to as “Before”, or B), (2) those which are already surface colonized, EPS-producing, and multi-celled entities, in other words, intact biofilm (*i.e.*, “During”; D), and also (3) those which have been subject to some degree of chemical degradation of EPS or physical disruption of a biofilm as a whole, the latter, e.g., as equivalent to the scraping of biofilm off of a surface (“After”, meaning following loss of biofilm integrity; A). Antibacterial actions are additionally distinguished into “Offense” (abbreviated as ω, *i.e.*, “Omega”) *versus* “Defense” (δ, *i.e.*, “Delta”). These respectively are efforts by antimicrobial-producing organisms to acquire resources *versus* efforts by antimicrobial-producing organisms to protect already obtained resources, such as colonizable surfaces as a resource, or “Space” more generally [33]. See Table 1 and Figure 1 for summary, the following paragraphs for discussion, and Section 3 for specific examples.

**Table 1:** Proposed categories of anti-biofilm activity. “Before”, “During”, and “After” refer to the biofilm state of “Target” organisms which are being impacted by antibiotic action. “Use in Defense” and “Use as Offense” refer to the utility of these agents to producers. Note that no reference is made within these categories of the degree to which a biofilm has matured following its initiation; degrees of “During” in other words are not considered. Abbreviations are provided for subsequent reference, e.g., “δB” stands for “Defense Before” meaning antibiotic-mediated *Defense* (δ) against disseminating bacteria, thus acting on target bacteria *Before* (B) they have formed biofilms. Such actions could be mediated, in this case, by either planktonic or biofilm-associated organisms. See also Figure 1 for a graphic summary.

State of Targeted Biofilm (below):	Use in Defense (δ) (resource protection)	Use as Offense (ω) (resource acquisition)
“Before” (B) biofilms have formed as the target state	δB: Protection of antibiotic-producing organisms from death or displacement that may be mediated by target, disseminating bacteria (ωD-1 or ωD-3 represent what potentially is being protected against)	ωB: Destruction of target, disseminating bacteria in order to obtain nutrients that are directly associated with those bacteria (δD-1 could serve as a potential counter measure mediated by these target bacteria)
“During” (D) biofilm sessile existence as the target state	δD-1: Protection of antibiotic-producing disseminating organism from target, biofilm bacteria (ωB represents what potentially is being protected against); δD-2: Protection of antibiotic-producing organisms as found within biofilms from encroachment or consumption by adjacent, target, biofilm bacteria (ωD-2 or ωD-3 represent what potentially is being protected against)	ωD-1: Displacement of target, biofilm bacteria by disseminating, antibiotic-producing bacteria (in order to obtain “Space”); ωD-2: Encroachment by antibiotic-producing, biofilm bacteria on adjacent, target, biofilm bacteria (in order to obtain “Space”); ωD-3: Destruction of target, biofilm bacteria by antibiotic-producing organism in order to obtain nutrients from those target bacteria
“After” (A) biofilms have been disrupted as the target state	δA: Destruction of target bacteria that have been displaced from biofilms, in order to prevent competition for nutrients	ωA: Destruction of target bacteria that have been displaced from biofilms, in order to obtain nutrients from those bacteria

Antibiotics may be useful as a defense (δ), particularly against still-disseminating bacterial invaders, that is, bacteria as targets “Before” (B) they form into biofilms; δB, Table 1. Antibiotics potentially also may be used as a defense against the expansion of unrelated, adjacent, target bacteria that are found within intact biofilm (“During”; δD-2). To the extent that fungi are capable of disrupting substrate upon which biofilms have formed, then antibiotics may be used either defensively (δA) or offensively (ωA) to impact target biofilms “After” those biofilms are no longer fully intact. Though not microorganisms, animals can also negatively impact biofilms. This in part they accomplish “After” (e.g., ωA) by first physically disrupting target biofilms such as via scraping and then digesting the now-removed material, though the scraping itself would count as “During”. More generally, such “After” actions by animals can be viewed as an offense (ωA) rather than defense (δA) to the extent that anti-biofilm activity is initiated to gain new resources, such as nutrients as resources, rather than to protect already obtained resources. Actions such as these would be in contrast to an organism protecting itself from invasion or attack. The action of immune systems against target biofilms or biofilm-forming bacteria similarly would count predominantly as defensive, δD.

To summarize, “Before” refers to a bacterial state prior to biofilm formation, “During” to bacteria that are present within intact biofilms, and “After” to bacteria that are associated with biofilms that have been disrupted by some non-bacterial factor, e.g., such as following fungus-mediated disruption of the substrate upon which biofilms have formed. Of importance, “Before”, here, is considered to refer particularly to bacteria that display relatively active metabolisms. “During”, by contrast, describes bacteria that display a variety of metabolic states, ranging from actively replicating and metabolizing to bacteria displaying much less active metabolisms and which likely are not replicating. Biofilm bacteria in particular tend to display heterogeneous physiologies within otherwise equivalent populations and especially relative to the more homogeneous physiological states that tend to be associated with bacterial populations found within well-mixed broth cultures. Bacteria physiological states associated with biofilms following disruption, *i.e.*, “After”, can by contrast be posited to potentially undergo a transition where less metabolically active bacteria become more metabolically active owing especially to their full or partial release from the now disrupted biofilm.

### 2.2. Limitations on Antibiotic Anti-Biofilm Activity, a Genetics Perspective

The first consideration in assessing the ecological utility of antibiotics as anti-biofilm agents is that it is possible that antibiotics do not serve the producers of antibiotics primarily as antibacterial agents (see the introduction to this section). In this case we could view the phenotypes associated with antibiotic expression collectively as pleiotropies, ones in which enhancement of proposed antibiotic non-antibacterial activities—an aspect of bacterial phenotype, that is, a trait—could come at the expense of antibacterial activities (a differing trait). The result is what can be described as an *antagonistic* pleiotropy, where the optimization of one function occurs at the expense of another function. This concept was originally formulated from the perspective of organism senescence, “*Genes that have opposite effects on fitness at different ages*,” (Williams [45], p. 400). Subsequently, it has become common to use the idea of antagonistic pleiotropy to describe conflicting impacts of mutations on organism fitness given an organism’s presence in differing environments [46,47], though the term is applied more generally as well [48]. Here it is the impact of mutations on antibacterial *versus* non-antibacterial activities that are posited to potentially “have opposite effects”.

It is conceivable that optimization of an organism’s non-antibacterial use of antibiotics could result in antibiotics that are less effective as antibacterials and/or, as is the emphasis here, less effective as anti-biofilm agents. Potentially contradicting this idea, Bleich *et al.* ([49], p. 3090) suggest that “*the structural complexity of many secondary metabolites makes it conceivable that they may similarly exert more than one biological effect*.” While it certainly is true that individual molecules can give rise to multiple biological effects, just as individual hormones in multicellular organisms can bind to different receptors, resulting in different cellular responses, it should be kept in mind that an ability to give rise to multiple biological effects should not necessarily be equated with all of those biological effects having been equally optimized by evolution.

We can extend this idea of antibiotic activity as pleiotropic to consider the antibacterial targeting of planktonic bacteria *versus* the targeting, instead, of biofilm-associated bacteria, with each as a distinct trait. Again, if targeting bacteria as found in one state represents a primary activity, then evolutionary enhancement of that activity could come at the expense of antibiotic ability to target bacteria that are present in different states—or, at least, evolutionary enhancement of elimination of bacteria that are found in these alternative states may not be simultaneously emphasized. For example, targeting emphasis could be on planktonic or more rapidly growing bacteria *versus* biofilm-associated or less rapidly growing bacteria.

An alternative but related possibility is that the process of antibiotic selection as pharmaceutical agents historically has been biased towards the development of antibiotics that are more effective against rapidly growing or planktonic bacteria [29]. Therefore, agents may have been selected for development which were less effective against bacteria displaying the particular physiologies that are found in biofilms. The trait in this case is artificial, the potential for drug commercial development, but to the extent that there is antagonism between antibiotic utility against planktonic bacteria *versus* biofilm-associated bacteria then there could as well be antagonism between historically perceived suitability for commercial development and effectiveness against biofilms.

## 3. Scenarios of Antibiotic Anti-Biofilm Ecology

A key consideration is whether we should expect natural selection to favor organism variants which happen to produce antibiotics that, given physiological or genetic constraints on effectiveness, can still substantially disrupt especially mature biofilms. This would be particularly antibiotics as anti-biofilm agents that are effective when used in isolation of other factors, as equivalent to how antibiotics often are used medically as antibacterial drugs. The resulting analysis is performed particularly from an ecological perspective.

Antibiotic-producing microorganisms are often isolated from soils [5]. Soils, as spatially structured environments with numerous surfaces, potentially possess large amounts of biofilm-associated bacteria [50]. We may expect therefore that soils would be an ideal location within which the production of effective anti-biofilm antibiotics might evolve. Why then are antibiotics that are isolated from soil-associated organisms typically not highly effective as anti-biofilm agents? As noted, one answer is that antibiotics used against biofilms may have been biased during their pharmaceutical selection against those that happen to possess substantial anti-biofilm activity. The evolution of substantial anti-biofilm activity in a single, selectively toxic, especially relatively small molecule furthermore may not be highly likely in terms of the ecological context of such evolution. To address especially the latter issue—of the possible presence or absence of ecological utility of antibiotics as antibacterial agents to the producers of these compounds—we can posit scenarios for antibiotic action against bacteria, such as in soils. These scenarios differ in terms of the properties of interacting individuals, biofilm-associated *versus* disseminating, which are either producing antibiotics or instead are serving as antibiotic targets. Also considered is biofilm disruption which initially is independent of antibiotic action. See Table 2 for reference to the various ecological scenarios explored.

Note that these scenarios effectively are thought experiments, with an assumption that organisms that are designated as antibiotic producing are actively producing as well as releasing antibiotic. In actuality, however, organisms do not necessarily produce antibiotic at the same levels throughout their life cycles. Thus, for example, actively metabolizing, motile, disseminating bacteria likely produce antibiotics at lower rates than bacteria that are entering stationary phase [1,18]. Nonetheless, rather than asking whether in the real world an organism in fact would be producing and releasing antibiotics under the circumstances considered, instead what is being asked, at least initially, is if antibiotics are produced then whether they might provide a utility to the producing organism. In particular: What is the potential that an antibiotic on its own may be useful to the producing organism towards killing and/or removal of established biofilms?

All of these scenarios can be viewed, at least in part, as descriptions of what can be defined as contest competition or, perhaps more familiarly, interference competition, which is competition [33] (pp. 20–21 for the following three quotations) that “*involves direct, antagonistic interactions between competitors, with the ‘winner’ appropriating the resource*…”. The objective, as primarily considered here in terms of anti-biofilm action (*i.e.*, “During”), is “the active displacement of existing colonizers…”. Addressed in particular is the question of whether “Clearing a space to colonize by eliminating prior residents can be accomplished by the production of antimicrobials”, and specifically by individual antibiotic production alone.

**Table 2:** Scenarios of antibacterial action based on the size of involved populations, presented as a 2 × 2 matrix. Numbers refer solely to the order of discussion in the main text. Parentheticals refer to the state of target organisms vis-à-vis biofilm status; see Table 1 for review. “Producing” refers to antibacterial production, e.g., such as the production of antibiotics, whereas a “Target” is an antibacterial-sensitive bacterium. Not indicated is Scenario 5, which instead involves antibiotic impact on target bacteria only “After” the biofilm they are associated with has by some alternative mechanism been disrupted. Abbreviations refer to “Defensive” (δ) or “Offensive” (ω) as well as “Before” (B) or “During” (D), *i.e.*, as employed in Table 1.

	One Target Cell	Many Target Cells
One Producing Cell	Not involving biofilm: Scenario 3 (“Before”, δB or ωB)	Effecting biofilm invasion: Scenario 1 (“During”, ωD, but also δD)
Many Producing Cells	Effecting biofilm protection: Scenario 2 (“Before”, δB, but also ωB)	Within-biofilm competition: Scenario 4 (“During”, δD or ωD)

### 3.1. Scenario 1, Disseminating Antibiotic-Producing Cell, Biofilm Bacteria as Targets

In the first of these scenarios we can envisage an antibiotic-producing bacterium which is in the disseminating stage of its life cycle. Particularly, this is a potentially biofilm-producing bacterium which is seeking a surface to colonize. If the bacterium encounters an already bacteria-colonized surface then this could represent a circumstance during which antibiotic might be deployed by the disseminating cell, or instead deployment of a non-antibiotic antibacterial substance [51]. The function of the antibacterial would be to locally clear the encountered biofilm (ωD-1, Table 1). Alternatively, and less stringently, the antibacterial in some capacity could facilitate the invasion of existing biofilm so that subsequent colonization, *i.e.*, biofilm formation by the antibiotic-producing bacterium might take place. In either case, this is production of antibiotic by a disseminating bacterium to facilitate biofilm invasion and subsequent biofilm formation by that same bacterium. Such biofilm invasion and subsequent formation need not always be effective to still on average be useful to the antibiotic-producing bacterium. Nevertheless, is the scenario feasible at all? In other words, can antibiotic produced by solitary bacteria give rise to at least localized biofilm elimination without other factors also playing a role in these processes, mechanisms not necessarily directly available to, for example, a physician treating a chronic bacterial infection?

#### 3.1.1. Ineffectiveness of Small Invading Forces

For the sake of visualization, consider a single antibiotic-producing cell that has become associated with the surface of a target biofilm. Antibiotic production by that cell could serve as a means by which it is able to initiate the process of subsequently forming a biofilm in an otherwise already biofilm-occupied location. A key issue in this scenario is that individual bacteria presumably are limited in the quantity of antibiotic that they can produce, and presumably are also not well equipped, as individual, otherwise non-predatory cells, to concentrate an MIC of that antibiotic within the immediate vicinity of target bacteria, much less through the many layers of bacteria that can make up the thickness of a biofilm. Specifically, antibiotics as soluble agents would be released by producing cells in all directions rather than focused in the direction of target organisms unless there is some mechanism to prevent such randomly oriented release. These two issues together—lone cells in combination with limits on the production and ability to locally concentrate antibiotics by those cells—potentially gives rise to a specific case of a more general consideration by Hibbing *et al.* [33] (p. 21): “*Highly motile organisms will be more likely to encounter potential competitors as individuals rather than in the context of a population of closely related organisms, thereby restricting their options for competitive strategies*.”

An analogy to the problems associated with one cell attempting to use antibiotics to clear many is a military dictum that an army requires a numerical advantage of soldiers, all else held equal, to possess a tactical advantage against an enemy [52]. For example, from Sun Tzu [53] (translation, p. 111, emphasis added): “*It is the rule in war, if our forces are ten to the enemy’s one, to surround him;* if five to one, to attack him; *if twice as numerous, to divide our army into two*.” Equivalently are what are known as Lanchester's Laws of military combat [54], with antibiotic release by a producing organism equivalent to the random, without aiming firing of guns or shooting of arrows (action-at-a-distance weaponry), thereby potentially conforming to Lanchester’s Linear Law [52]. This Linear Law represents a best case scenario for an out-numbered but otherwise per capita equivalent force since it posits that the disadvantage to the smaller force is no greater than the difference in number between the two forces. Thus, a single cell “Firing” antibiotic randomly at an entrenched “Army” of tens, hundreds, or thousands of target bacteria would not necessarily succeed in displacing those bacteria, and particularly this would be the case to the extent that the target bacteria are capable of “Fighting” back in some manner with equivalent per-cell capacity (*i.e.*, facilitation of δB, Table 1; see equivalently Scenario 2, below; see also [55]). This is a problem [52] (p. 57) of the “impotence of small forces in the presence of one of overwhelming power”.

#### 3.1.2. Insufficiency of Soluble Antibacterials as Facilitators of Biofilm Invasion

Also consistent with the models of Lanchester [52], an organism explicitly would need to display a greater “Fighting” capacity in order to reach parity with more numerous opponents, but is this likely based on antibiotic production alone? Part of the answer to this question, in the negative, is physiological. In particular we can question the assumption of Scenario 1 that antibiotic is produced by disseminating bacteria. For at least some bacteria, antibiotic production occurs in response to quorum sensing, e.g., [18,33], which is at least suggestive that antibiotic would not be produced by solitary disseminating cells. Antibiotics also tend to be produced by bacteria as they enter into stationary phase, rather than during exponential phase [18], and therefore again presumably antibiotic production is not a feature of metabolically active disseminating bacteria. The very concept of secondary metabolites in fact has historically been defined for microorganisms as those compounds that are produced particularly [56] (p. 71) “at late stages of microbial growth…”.

Experimental evidence appears to support this idea that disseminating bacteria may be poorly equipped, based on the use of soluble antibacterial substances alone, to effect at least the initial displacement of established bacterial biofilms. Tait and Sutherland [57], for example, looked at the ability of planktonic, antibacterial-producing bacteria, here bacteriocin producers, to facilitate initial stages of biofilm invasion. The bacteriocin producers, however, seem to have been no more effective against sensitive biofilms than non-producers. With time, though, these factors appear to become more useful, which can be interpreted as a probable utility associated with antibacterial production *following* successful invasion and colonization, *i.e.*, Scenario 4, *versus* antibacterial-mediated clearing of biofilm bacteria during the initial stages colonization (Scenario 1). In other words, invasion initially appears to be successful in these experiments with or without potential bacteriocin activity. Though subsequent competitive success may be attributable to antibacterial production, we can speculate that this success involved factors other than just antibacterial production, e.g., such as, at a minimum, replication of the invader. So far as one can tell in these experiments, a single cell does not appear to have invaded an established biofilm by using soluble antibacterial agents to first eradicate the biofilm, that is, such that surface attachment and then subsequent growth by the invader could occur.

Consistent with the idea that invasion by solitary cells into biofilms need not be dependent on the production of soluble antibacterial substances, Houry *et al.* [58] found that flagellated bacilli were able to invade mature biofilm matrix perhaps as a function of the kinetic energy associated with swimming cells alone. That same study also provides an example of how antibacterial activity alone can fail to remove biofilms—in this case lysostaphin treating *Staphylococcus aureus* biofilms—but can succeed in combination with other mechanisms, which in this study involved penetration of producing cells into the targeted biofilm. Invasion of solitary cells into mature biofilm thus appears to precede antibacterial action rather than the converse.

In addition to the problem of disseminating cells not necessarily producing antibiotic, target biofilms can be more antibiotic tolerant than planktonic bacteria. As appears to be seen with some prominence clinically, the close proximity of bacteria and/or their slow growth [59,60] within established biofilms tends to generate antibiotic tolerant persister phenotypes [36,37]. Moreover, multiple species can be present within naturally occurring biofilms and these can vary in their antibiotic sensitivity and/or can enhance each other’s resistance [50,61,62,63,64].

Altogether, this potential for biofilm-associated bacteria to display antibiotic resistance or tolerance, and even to produce antibiotics (Scenario 2, below), Lanchester [52] (p. 57) might have described as being “dug in”, which is to have established a defensively fortified fixed position. Indeed, Jefferson [65] has argued that one of the reasons that microorganisms produce biofilms could be explicitly for “Defense”, though the evidence provided in that publication is biased towards the medical rather than the environmental. Matz [66], consistently, described biofilms as a “refuge against predation”, in this case, to a degree, inhibition of phagocytosis by protists. Furthermore, biofilm maturation might serve generally as a means of resisting antagonistic interactions with other bacteria [55,67]. Collectively, mechanisms of resistance or tolerance to the action of antibiotics may give rise to further reductions in the potential of lone bacteria to displace or invade existing bacterial biofilm via antibiotic production alone.

#### 3.1.3. Requirements for Effective Biofilm Invasion and Displacement

The invasion and then displacement of existing biofilm, particularly by disseminating or equivalent bacteria deploying antibacterial agents, has been observed, though without providing evidence for invasion or displacement success based on antibiotic production alone. Hibbing *et al.* [33], for example, note that bacteria displaying swarming motility, e.g., *Myxococcus xanthus*, can encounter target bacteria as groups, which are described as “wolfpacks”, and they thereby may be able to circumvent the problem that individual disseminating cells can be wanting in competitive ability. Xiao *et al.* [68] furthermore provide evidence that *M. xanthus* employs what they describe as “secondary metabolite antibiotics” as a necessary component of laboratory predation of *Escherichia coli*. In the laboratory, *M. xanthus* as individual cells is even able to lyse target bacteria as well as consume bacterial microcolonies [69] (ωD-3, Table 1). They appear to achieve such lysis, however, following a combination of inserting themselves into the immediate midst of target bacteria, e.g., as equivalent to as seen with the experiments of Houry *et al.* [58], and then producing additional antibacterial factors, particularly digestive compounds; see also [70]. The *Myxococcus*-attacked laboratory microcolony, however, was small, consisting of only about 20 cells. Furthermore, it has been hypothesized that *M. xanthus* might induce prey bacteria to lyse only upon cell-to-cell contact, delivering multiple antibacterial substances directly to targeted organisms, assuring that “*expensive secondary metabolites are not lost through diffusion*” ([71], p. 8). *Bdellovibrio bacteriovorus* as well as other, similar bacteria also appear to be able to display such contact-dependent epibiotic predation of bacterial prey [72].

Planktonic cells of the marine bacterium, *Pseudoalteromonas tunicate*, also have been shown to displace preexisting biofilms. Here, overnight cultures of 10^6^ cells/mL were incubated in contact with target biofilms under static conditions for one hour. The resulting displacement of biofilm bacteria, however, required production of a 190-kDa, potentially cell-surface-associated antibacterial protein [60] as well as other antibacterial substances [67] rather than being effected exclusively via the use of soluble, more antibiotic-like agents; see also [51]. Al-Bakri *et al.* [73] found that 48-h old biofilms of *Burkholderia cepacia* were somewhat displaced following challenge with *Pseudomonas aeruginosa* cells in numbers that were on the order 1/1000^th^ to 1/100^th^ those of *B. cepacia*. The *P. aeruginosa* strain produced a *B. cepacia* growth-inhibiting substance, but declines in *B. cepacia* numbers did not occur until bound numbers of *P. aeruginosa* reached approximate parity with *B. cepacia*. Such apparent invader colonization prior to biofilm displacement is suggestive that this is an example of Scenario 4 rather than Scenario 1 (Table 2); see equivalently Tait and Sutherland [57], as discussed above. A role for additional factors in biofilm displacement cannot be ruled out as a second *P. aeruginosa* strain that did not equivalently produce a *B. cepacia* growth-inhibiting substance was also able to displace *B. cepacia* biofilm, though not to the same degree. Lastly, there are experiments documenting bacterial invasion of existing biofilms where this ability has not been attributed to antibacterial action [51,57,74], suggesting at a minimum that mechanisms other than the production of soluble antibacterial agents, such as antibiotics, potentially play roles at least experimentally in abetting such invasion.

Aggressor bacteria may bring to bear superior numbers, multiple or non-antibiotic antibacterial agents, and indeed may directly target bacteria in the course of cell-to-cell contact. Under these conditions, intact biofilms may indeed be susceptible, at least locally, to invasion and/or eradication by one or more disseminating individuals. Biofilm bacteria thus can be invaded as well as be severely impacted by motile or planktonic bacteria, but evidence that this can be achieved solely via the production of a single antibiotic type by individual bacteria does not, to the best of my knowledge, appear to exist. It may also be possible to circumvent the issue of individual cells not necessarily being able to generate MICs of specific, biofilm-eliminating *antibiotics* by their instead employing alternative, single-hit killing, soluble antibacterial agents [75,76]. Such agents include tailocins, or, if the attacking cells are lysogens, then temperate bacteriophage virions [77], with the latter process colorfully dubbed “Kill the relatives” by Paul [78]. In practice, however, a producing cell must lyse to release these single-hit killing agents and an *individual* producing cell consequently cannot simultaneously release these agents and ecologically compete [79,80,81].

Overall, then, antibiotic utility as a stand-alone means of displacing entrenched biofilms from colonized surfaces, for the sake of an individual microorganism invading an already occupied surface niche, *i.e.*, Scenario 1, does not appear to be well supported either logically or empirically. Biofilm invasion by aggressor bacteria further appears to be possible without, so far as one can tell, antibacterial release. Soluble antibacterials, especially acting alone, in particular do not appear to serve as effective anti-biofilm agents except *following* biofilm invasion rather than causing such invasion. The implication is that additional mechanisms beside antibiotic production—resulting in or allowing for invasion into biofilms along with subsequent colonization, with the latter as gives rise to Scenario 4—likely are required for individual disseminating bacteria to subsequently kill or clear mature biofilms in the course of antibiotic action.

### 3.2. Scenario 2, Antibiotic-Producing Biofilm, Disseminating Target Bacterium

In the second scenario a single target cell encounters an established, antibiotic-producing biofilm. That an established biofilm might inhibit invasion by a disseminating bacterium via the production of antibiotic that targets that bacterium (δB, Table 1) is at least plausible given the high numbers and concentrations of bacteria making up biofilms. Production of antimicrobial substances by biofilm-associated bacteria also has been demonstrated [82,83], including in sufficient quantities by single-species biofilm to inhibit the growth of invading sensitive bacteria [50,73,84]. Indeed, the potential for bacteria-derived compounds to inhibit the formation of biofilms would appear to be beyond dispute [85]. Whether biofilm-produced antibacterials are always effective at preventing invasion by even sensitive bacteria, however, is an open question. Tait and Sutherland [57], for example, found that sensitive bacteria could readily establish themselves in biofilms among bacteriocin-producing bacteria to which they were sensitive.

Notwithstanding these issues of potential inefficacy, and contrasting Scenario 1, Scenario 2 is one in which antibiotic production is used defensively against a bacterium “Before” that bacterium has had an opportunity to successfully colonize a surface. Consequently, this is *not* a scenario of biofilm clearance so much as one of biofilm prevention. Scenario 2 therefore does not consist of antibiotic deployed to disrupt intact biofilm. See Nadell *et al.* [86] for a similar anti-colonization function but attributed to EPS. At least arguably similar, and occurring explicitly in association with soil, is the deposition of antibiotic-producing streptomycete predominantly into the outer layers of beewolf larval cocoons [87]. There the resulting antibiotics conceivably also serve anti-microbe colonization functions. Antibiotics thus may serve defensive functions, and perhaps particularly as mediated by biofilms. There is little reason to expect, however, that this specific ability might translate directly into the evolution of antibiotics that possess a capacity to eradicate mature bacterial biofilms nor, particularly, antibiotics that are able to accomplish this anti-biofilm function independent of additional mechanisms.

### 3.3. Scenario 3, Disseminating Antibiotic-Producing Cell, Disseminating Target Bacterium

The third scenario involves encounter of individual antibiotic-producing cells with individual target bacteria. As with Scenario 1, antibiotic-producing cells other than epibiotic predators are unlikely to intrinsically possess an effective means of concentrating antibiotics within the vicinity of encountered cells, particularly motile cells. Target organisms nevertheless may be somewhat susceptible to antibiotic action given that they are actively metabolizing, as too should be the case for Scenario 2. At the same time, however, actual disseminating bacteria, as non-predatory individuals that are fairly metabolically active, potentially are less likely to be producing antibiotic (see discussion, Section 3.1.2).

Scenario 3 thus is likely not highly relevant to understanding the ecological utility of antibiotic production for perhaps most producing bacteria. It nonetheless serves as a contrasting scenario to phage action against planktonic target bacteria, or indeed to the action of predators of planktonic bacteria generally, e.g., bdellovibrios [70,88]. That is, the issue of producing sufficient densities of antibacterial substances within the vicinity of target bacteria—the “Concentration” [52] of antimicrobial weaponry against a single target—may be more effectively addressed by organisms that are able to deliver antibacterial agents directly to the interior of target cells. It is helpful also for antimicrobial delivering organisms to move in concert with target organisms, e.g., as is the case for phages in the course of infection of motile bacteria, since infecting phages are found inside of these moving organisms. Alternatively, disseminating bacteria, if they have successfully attached to a surface, may begin to replicate to form biofilms. At this point they may be able to more effectively deploy antibiotics against encountered or adjacent bacteria, thereby potentially generating Scenario 2, as well as Scenario 4.

### 3.4. Scenario 4, Antibiotic-Producing Biofilm, Biofilm Bacteria as Targets

The fourth scenario, like Scenario 1, again considers antibiotic utility “During”, that is, as used against intact biofilms, though like Scenario 2 these antibiotics are also as produced *by* biofilm cells. Consistent with these dual parallels, antibiotics in terms of Scenario 4 may be viewed as serving in both offensive (ωD-2 or ωD-3, Table 1) and defensive (δD-2) capacities, with antibiotics acting as a component of the “Warfare” that presumably can occur among the heterogeneous organisms that can make up biofilms. Biofilms consisting of more than one species likely are very common and Elias and Banin [62] along with Rendueles and Ghigo [62] review, respectively, the cooperative and antagonistic properties of these mixed or multi-species biofilms. See also Moons *et al.* [88]. In terms of defensive actions, Hibbing *et al.* [33] (p. 21) note that “*Once a bacterium or bacterial population is established at a favourable location, long-term* *persistence requires mechanisms for preventing encroachment by potential competitors*.” Offensively, Nadell *et al.* [86] suggest that “*competing strains attempt to displace one another from occupied substrata*…”. In Scenario 4, both of these processes may occur in the guise of competition between the individual genotypes making up biofilms [55,89].

#### 3.4.1. Evidence of Within-Biofilm Antibacterial Effectiveness

In terms of experimental evidence for Scenario 4, especialy ωD-2 (Table 1), Tait and Sutherland [57] provide just such a scenario of successful within-biofilm antagonism, though involving bacteriocins rather than antibiotics. In that study the bacteriocin producers displayed a competitive advantage within mixed biofilms over sensitive bacteria. In a more recent study, Rendueles *et al.* [90] identified an *E. coli* strain that produced a bacteriocin only during biofilm growth. The antibacterial agent also was more effective against biofilm-associated bacteria than planktonic bacteria plus, as a consequence of bacteriocin production, was able to outcompete sensitive bacteria more effectively within mixed biofilms.

Yan *et al.* [82] found that the antibiotic bacitracin was produced by a strain of *Bacillus licheniformis* within biofilms but not by planktonic cultures, and they suggested that antimicrobial production could contribute to a bacterial strain’s domination of mixed biofilms. Yan *et al.* also hypothesized that such antimicrobial production primarily by biofilms may be generalizable to other bacterial types; see also as reviewed by Prol García *et al.* [89]. Moons *et al.* [91] observed elimination of *E. coli* within mixed biofilms by an antibacterial-producing strain of *Serratia plymuthica*, but there was a lack of elimination following co-culture with equivalent strains knocked out in terms of antibacterial production. The starting ratio of *S. plymuthica* to *E. coli*, however, was 100 to 1 in favor of the *Serratia* strains.

In the experiments of Al-Bakri *et al.* [73], declines in *B. cepacia* presence did not occur until *P. aeruginosa* biofilm numbers had become elevated to equivalent levels within mixed-species biofilms. This suggests that competition was occurring between somewhat established members of a mixed biofilm rather than between an established member (*B. cepacia*) and a disseminating bacterium (*P. aeruginosa*). See as well Schluter *et al.* [92] who also considered intra-biofilm competition, as equivalent to Scenario 4, ωD-2 (Table 1), though with EPS serving as the competitive factor rather than soluble antibacterials. Overall, then, antibiotics produced within established biofilms in principle might protect individuals from being displaced by adjacent, also established bacterial aggressors. Antibacterial agents certainly seem to be able to at least contribute to the ability of aggressors to effect such displacement.

#### 3.4.2. Does Killing or Removal of Biofilm Bacteria Occur via Antibacterial Action Alone?

Likely key to the ability of bacteria employing soluble antibacterials to outcompete sensitive bacteria, as observed in the above-noted studies, is the close physical association that can be enforced between antibacterial producers and sensitive bacteria within mixed biofilms. This issue appears to be exemplified by the results of Rendueles *et al.* [90]. They found that the ability of bacteriocin-producing strains to completely displace sensitive strains declined as the fraction of producing cells within mixed biofilms also declined, *i.e.*, suggesting that increased spatial distance between producers and targets [57] reduced the ability of bacteriocin to fully penetrate to sensitive bacteria (see especially their Supplementary Figure S11 [90]). In addition, it is uncertain from these experiments whether production of soluble antibacterial substances alone contributes to an outcompeting of sensitive bacteria within biofilms or whether additional mechanisms are at play even given competition between otherwise equivalent strains, e.g., such as EPS-mediated displacement. Particularly, metabolically active bacteria might be able to pry otherwise identical but metabolically inactive bacteria off of surfaces solely because one strain is able to grow and replicate whereas the other is not.

A second issue is that not all antibiotics are bactericidal, and of those antibiotics that are bactericidal, not all actively lyse target bacteria. Lysis may be especially an issue in terms of the displacement of antibiotic-sensitive bacteria that have become highly immobilized within EPS [93]. Removal of established biofilms may be particularly difficult if bacteria that are directly bound to surfaces are also less metabolically active [59], e.g., to the extent that these bacteria are deprived of nutrients or oxygen [94], and as a result are more likely to display persister phenotypes. Consequently, even if an antibiotic-producing bacterium can interfere with the metabolism of a neighbor, how efficiently can that antibiotic-mediated interference translate directly into sufficient clearance of those bacteria from surfaces such that displacement and re-colonization might take place? Indeed, how effective might antibacterial-producing strains be at outcompeting sensitive bacteria without some means by which antibiotic-producing aggressors can move towards or physically penetrate into groups of adjacent, sensitive bacteria—including via cell division by the aggressor—so that soluble antibacterial might be concentrated to MICs within the immediate vicinity of target bacteria?

An additional issue is the nature of many mixed-biofilm experiments such that a substantial amount of observed competition likely is between actively growing bacteria rather than antibacterial activity against bacteria that are found within less metabolically active biofilms or regions of biofilms. Such mixed-biofilm experiments thus may be biased towards displaying an effectiveness of soluble antibacterials as competitive agents that would be less apparent given more mature biofilms as targets. It is especially against persister-containing, more-established biofilms that antibiotic therapy is less effective, e.g., such as likely are seen with chronic bacterial infections [1,17,60,95,96]. Ultimately, therefore, it is difficult to tell whether demonstration of an ecological utility in mixed-biofilm experiments, as reviewed in the previous section, is evidence that antibiotic-producing organisms use antibiotics as a means of substantially impacting especially mature bacterial biofilms.

This issue of target biofilm maturity may be addressed by using invasion assays, such as those of Tait and Sutherland [57] or Al-Bakri *et al.* [73]. Again, however, it is not certain that the production of soluble antibacterial agents alone in these experiments is what gives rise to displacement of sensitive bacteria nor whether allowing for greater maturity of target biofilms with these assays would result in greater resistance—Tait and Sutherland employed 3-day-old 30 °C-grown biofilms while Al-Bakri *et al.* used 7-day-old 37 °C-grown biofilms. In addition, displacement of sensitive bacteria usually is not 100% and it is likely that here too a key to anti-biofilm success is close physical proximity between antibiotic producers and antibiotic targets in combination with some means of aggressor movement towards or into groups of target bacteria. Thus, despite the evidence presented that soluble antibacterials apparently can contribute to the competiveness of producers relative to sensitive bacteria, there remain numerous questions about the potential of antibiotics acting alone to serve antibiotic producers, in nature, towards even local eradication of mature, otherwise genetically antibiotic-sensitive biofilms.

#### 3.4.3. The Issue of Public Goods

Antibiotic action could lead to the exploitation of target bacteria as nutrients (ωD-3, Table 1). Antibiotic-producing organisms tend to be nutrient absorbers, however, so it is only if at some point target cells are extracellularly digested that consumption by antibiotic-producing organisms may occur. Might benefits of such nutrient acquisition, were it to occur, nonetheless result in the evolution of an antibiotic activity which is capable, acting independently of other factors, of killing or clearing mature bacterial biofilms? One issue here is that if antibacterial-generated nutrients become soluble so that subsequent absorption can take place, then what is to stop unrelated, non-antibiotic-producing organisms from utilizing those nutrients as well? Or what is to stop other organisms from colonizing surfaces that have been cleared, hypothetically, also by antibiotic action? More generally, how do antibiotic-producing organisms that are otherwise found in biofilms deal with the “Public good” [97] costs of producing and releasing soluble antimicrobial factors that have the potential to free up resources that may then be available to other organisms that do not produce these factors [98]?

Drescher *et al.* [99] present evidence of biofilm-based solutions to a public goods problem. These solutions are posited in terms of (1) within-biofilm nutrient generation, (2) retention of soluble agents within the EPS of producers, and (3) rapid removal of soluble agents from the vicinity of nutrient production due to fluid flow occurring outside of EPS. Specifically, the Drescher *et al.* model predicts that public goods will have utility predominantly to the producers of those goods particularly when the public good is able to concentrate around the producing cell but, explicitly, also *not* concentrate around non-producing cells. This scenario, however, is inherently unable to address the public goods problem associated with offensive actions as mediated through the use of antibiotics against neighboring cells within biofilms. Instead, the Drescher *et al.* solutions are the polar opposite of what is required for effective antibacterial use against organisms found adjacent to producing organisms within biofilms, which instead should involve a concentrating of antibiotic around the target, that is, non-producing organism, rather than primarily around the producer. The antibiotic as the public good, or public good generator, indeed must be retained close to the producer, but not so close that it fails to be retained also close to the target. With Scenario 4 in terms of offensive action (ωD-2 or ωD-3, Table 1), however, the antibiotic target likely will be found outside of the producer’s EPS rather than the producer concentrating the public good solely within the confines of its own EPS.

#### 3.4.4. What Works and What Doesn’t

An argument has been put forth that antibiotic-producing bacteria tend to be found within otherwise antibiotic-resistant populations of related bacteria [100]. Given that similar organisms tend to populate similar niches [55,98], and therefore that antibiotic-producers and related antibiotic-resistant conspecifics will tend cluster, then this could imply that a substantial fraction of interactions within established biofilms in nature could be between antibiotic-producing and antibiotic-resistant organisms rather than between antibiotic-producing and antibiotic-sensitive ones. Indeed, the latter interactions may tend to be short lived, perhaps involving especially Scenario 2, *i.e.*, δB (Table 1), where the antibiotic-sensitive organism never has the opportunity to join an already formed, antibiotic-producing biofilm. An additional issue is that if antibiotic-producing bacteria are able to persist within biofilms because they have attached to other bacteria [62]—or otherwise are usefully interacting with those other organisms within, for example, multi-species biofilms [64]—then what happens to antibiotic producers if those other organisms are harmed by the produced antibiotic? In short, there are multiple reasons for why antibiotic production within biofilms could fail to aid producers as weapons wielded against established members of the same biofilm.

In light of these issues, along with those raised for Scenario 1, it may be tentatively concluded that antibiotics serve especially as defenses by or within biofilms, particularly against invasion by sensitive bacteria, *i.e.*, Scenario 2 (δB, Table 1) but also the defensive aspects of Scenario 4 (δD-2). There, however, does not appear to be substantial experimental evidence for either utility. Alternatively, antibiotics may exist as only one component of multiple mechanisms that can be used as means of procuring resources from bacteria making up the same biofilm, e.g., EPS to pry bacteria off of surfaces [92] (ωD-2) or digestive enzymes to convert metabolically inhibited bacteria into soluble nutrients (ωD-3). At a minimum, it appears that antibiotic effectiveness as anti-biofilm agents might require delivery directly to the immediate vicinity of target bacteria, presumably to relatively high within-biofilm concentrations that likely must then be maintained as such over relatively long periods of time. Clinically, such circumstances may be precisely the means by which otherwise antibiotic tolerant biofilm bacteria are successfully treated: Generation of overall high antibiotic concentrations [16] and/or delivery of antibiotics directly to or, especially, into biofilms [101,102,103], thereby establishing high antibiotic concentrations especially within the immediate vicinity of target bacteria. The latter can be achieved particularly given some degree of forced penetration into extracellular matrix, and this may be accomplished in part via EPS disruption. EPS disruption can directly contribute to the physical removal of biofilm from surfaces as well.

### 3.5. Scenario 5, Antibacterial Action Following Biofilm Physical Disruption

Biofilm-associated bacteria can develop antibiotic *tolerance* [1,31] which is an antibiotic insusceptibility that is temporary, heterogeneously present across biofilms, and not associated with heritable allelic variation. One means by which such biofilm-associated antibiotic tolerance can be overcome is by disrupting a biofilm, e.g., [101,102], as can be accomplished via physical, chemical, or enzymatic action, or simply cell disaggregation [104]. For example, Houry *et al.* [58] found that antibacterial action could be substantially improved following the literal poking of holes into biofilm EPS by motile bacilli, holes through which antibacterial was then delivered by those same bacteria. To the extent that fungi can digest the substrate upon which biofilms have formed, then it is conceivable that antibiotics produced by the same fungi could similarly become more effective against the bacteria making up those biofilms. In this scenario, therefore, there is less of an impact via antibiotic production on still-intact biofilms (“During”) and more of an impact on no longer intact biofilms (“After”; Table 1).

Antibiotics as so expressed could be used defensively by inhibiting the functioning of potentially competing organisms (δA, Table 1). This may be competition for the same soluble nutrients that the antibiotic-producing organism is generating via its secretion of exoenzymes, enzymes which happen to degrade as well the substrate upon which biofilms have formed. In addition, though potentially requiring additional enzymes, the substrate-disrupting, antibiotic-producing organism might consume the antibiotic-affected bacteria as well (offensive function; ωA, Table 1). Note that the potential to eradicate biofilm-associated bacteria is presumed, in Scenario 4, to require more than just antibiotic action, *i.e.*, minimally, for example, substrate-disrupting exoenzymes are involved as well.

## 4. Bacteriophage Anti-Biofilm Activity

As considered above, antibiotic production on its own in most cases is probably insufficient to allow producing cells to effectively disrupt mature biofilms. Killing of biofilm bacteria, however, may be achieved especially given sustained concentration of antibiotic within the immediate vicinity of bacterial targets, as may be accomplished by a variety of mechanisms including as effected by antibiotic-wielding predators of bacteria. Predators of biofilm-forming organisms in addition appear to bring to bear further antibacterial strategies to disrupt as well as consume biofilms. These biofilm-consuming organisms can include animals [105] but also protists [66,106]. Microorganisms that are able to move into the midst of biofilms as well as deploy multiple disruptive factors can destroy and even obtain nutrients from biofilm bacteria, such as seen with the predatory bacteria, *M. xanthus* (above) or *Bdellovibrio bacteriovorus* [70], and this, we can speculate, could be the case for certain fungi as well. Another important predator of bacteria—a type of organism which is able to acquire, extract nutrient resources from, and then kill a bacterium—are bacteriophages.

### 4.1. Bacteriophages as Anti-Biofilm Agents

That biofilms can serve as targets of phage predation has been addressed by a number of reviews [107,108,109,110,111,112,113,114]. These references, however, primarily address the use of phages as applied anti-biofilm biocontrol agents. This section provides a brief overview of phage use as anti-biofilm agents. Phages in particular have been employed clinically to combat presumptively biofilm-associated chronic bacterial infections. For additional references and discussion of this phage-mediated biofilm biocontrol, see the companion article [19].

Phage therapy is the use of bacteriophages as antibacterial agents especially in clinical settings [3,115]. There exists a long history of phage use in roles that are equivalent to those of antibiotics, as well as ongoing medical use of phage therapy in the treatment of bacterial infections in humans [116,117,118]. Indeed, phage use as antibacterial agents predates the discovery of antibiotics. The use of phages as anti-biofilm agents can take place either for the treatment of bacterial infections or instead towards the removal of unwanted bacteria from extra-organismal environments.

Phage-mediated biocontrol of biofilms can involve either phage application prior to biofilm formation (*i.e.*, as equivalent to “Before”, Table 1), application to already formed biofilms (“During”), or indeed phage impact that is found in association with additional mechanisms of physical biofilm disruption (as equivalent to “After”). In all cases, at a minimum, phage virions must be applied in such a manner that they are able to reach target biofilms in sufficient numbers. This is just as antibiotic action similarly requires sufficient antibiotic amounts to achieve antibacterial efficacy. Phages, however, can be safer than antibiotics [119], are less likely to possess pharmacologically emergent properties [34], tend have less of an impact environmentally, and can also possess useful pharmacokinetic properties, particularly an ability to increase their numbers in association with their antibacterial activity [120,121].

The impact of phages on biofilms involves an initial bacterial adsorption step that is followed by bacterial infection. When employing phages that are obligately lytic, as generally is the case with phage-mediated biocontrol of bacteria, then phage infection results in both the killing of sensitive bacteria and their lysis. This likely both impacts biofilms structurally and releases new phage virions that potentially can reach and then infect adjacent bacteria [122]. The result is a cyclical acquisition and then killing of biofilm bacteria, though nevertheless which may require augmentation for effective biofilm removal via further external application of phage virions [19]. The removal of biofilm bacteria via lysis potentially results as well in physiological changes among formerly biofilm-buried bacteria, allowing those bacteria to more effectively support subsequent phage infection. This impact of phage-induced bacterial lysis in combination with associated phage population growth can be described as an active penetration of phages into bacterial biofilms [120]. In addition to the impact of phage lysis on the structure of bacterial biofilms, certain phages either naturally or following bioengineering can express EPS depolymerase enzymes. Given a good match between enzyme and EPS structure, then these EPS depolymerases can additionally contribute to the degradation of biofilm structure [123].

### 4.2. Biofilms as Targets of Phage Action

Bacteriophages in principle can interact with biofilm-forming bacteria offensively either “Before” biofilms form (ωB, Table 1), “During” biofilm formation and maturation (especially ωD-3), or following biofilm disruption (“After”, *i.e.*, ωA). Their potential to interact with biofilm bacteria at any one of these stages is a function of numbers of bacterial targets and the target size of individual bacteria as well as bacterial target susceptibility to phage adsorption. As phage host ranges tend to be fairly narrow [124], the number of target organisms with which a phage may interact within a given environment is not necessarily large. For phages that have specialized on biofilm-forming bacteria, the opportunities for interaction with target bacteria "Before" they have formed biofilms is probably somewhat lower due to a relative rarity of such bacteria in comparison to their biofilm-associated parents. See Abedon [125] for a general discussion of the likelihood of phages interacting with target bacteria that are present at low densities.

Unless phages are actively causing the physical disruption of biofilms, such as through the action of EPS depolymerases, or phages and biofilm destruction are both widespread within an environment, then phage interaction with biofilms “After” such disruption we also can speculate would be fairly rare. Instead, biofilm-forming bacteria could exist in environments predominantly as or within biofilms, in terms of total numbers of cells. Especially if those bacteria found within biofilms also exist as aggregations of closely related cells, ones that collectively present larger targets to bacteriophages than do individual bacterial cells [122], then we can predict that it is predominantly within existing biofilms, “During”, that phages interact with biofilm-forming bacteria. As argued below, bacteriophages may serve as effective biofilm disruptors in part because phages are able to efficiently concentrate their antibacterial activity both within and in the vicinity of target biofilm-associated bacteria. For further review of the potential dynamics of phage-biofilm ecological interactions, as well as phage use as biofilm biocontrol agents, see [19].

### 4.3. Concentrating Phage Antibacterial Activity

As discussed above, an outnumbered but otherwise equivalent attacking force inherently is unlikely to defeat an enemy. This is true, however, only to the extent that the two opposing forces are equivalently matched on a per-individual basis. Bacteria can possess anti-phage defenses and those defenses can be viewed as analogous to the immune systems of multicellular organisms [126]. To effect a “Tactical advantage” over numerous biofilm-associated, potentially hostile bacteria, phages therefore must engage in what can be described as forms of asymmetric or unconventional warfare, e.g., exploiting vulnerabilities of bacteria along with repurposing a bacterium’s “Infrastructure” to be used against that bacterium. Phage tactics also can be viewed as variations on the famous Trojan Horse strategy, the gaining of innocuous entry into a fortification which is followed by surreptitious bolstering of the attacker’s ranks. Lanchester [52] makes a similar point by noting that the overwhelming advantage of possessing a larger force (p. 56) “*manifestly does not apply to the case of a small force concealed*…”.

#### 4.3.1. Trojan Horse Strategy Number 1

The Greeks in the Trojan War were unable to defeat the city of Troy while attacking from outside of its walls but were able to do so, or so the legend goes, once they had conveyed soldiers secretly to within those walls, inside of the Trojan Horse. This concept of a Trojan Horse strategy as applied to phages has been used previously [127]. Specifically, it has been employed to describe phage-bacterial interactions where macrophage-associated bacterial pathogens, e.g., *Mycobacterium tuberculosis*, are targeted by phages that have been taken up into the macrophage inside of another, benign bacterium (*Mycobacterium smegmatis*). The bacterial “Trojan Horse” then lyses, releasing phages that are able to acquire and then destroy the now co-located intracellular bacterial pathogens [128].

In terms of Trojan Horse-mediated exploitation of a single bacterium by a phage, the “Horse” simply is the phage capsid itself, which the bacterium unwittingly allows to have access to its otherwise well-fortified, indeed “Walled” cytoplasm. The bacteriophage genome escapes from its capsid and then brings additional “Soldiers” into the cell in the form of antibacterial factors generated by coopting the protein expression machinery of its host, which the phage also uses towards its ultimate aim of producing progeny bacteriophages. Just as with the original Trojan Horse scenario, this tactic is effective because it is a means of concentrating soldiers, here phage-expressed antibacterial activity, precisely where they can have the greatest impact, which is within the bacterium itself.

This proximity between the source and the targets of antibacterial activity provides an important advantage that phages hold over most antibiotic-producing organisms. Specifically, antibiotic-producing cells that are located externally to their bacterial target should be much less able to equivalently concentrate especially soluble antibacterial activity on those targets. As an alternative perspective on this same idea, Curtright and Abedon [34] (p. 10) note that “*a phage virion in principle is simply a bacterium-acquisition devise whose sole function is the delivery of the intracellular acting agents to bacterial cytoplasms*.” (The Trojan Horse equivalently was also simply a Troy-acquisition device whose sole function was the delivery of Greek soldiers to within the walls of Troy.) Of importance, note that an ability to concentrate both antibacterial activity and resource generation within target bacteria also allows bacteriophages to avoid generating publically, particularly extracellularly available goods.

#### 4.3.2. Trojan Horse Strategy Number 2

The parallels between phage tactics and those exemplified by the Trojan Horse possess an additional layer, one which is applicable especially to the “Conquest” of groupings of related bacteria, particularly clonal bacterial microcolonies or cellular arrangements as can make up biofilms [122]. Here the Trojan Horse is the first of the cells that becomes phage infected within these cellular clumps. In this case the recruitment of additional soldiers is provided by phage replication within that cell, with the resulting “Battle” taking place immediately within the associated microcolony or arrangement. As equivalently argued in Section 3.4, antibiotic utility to producing organisms as anti-biofilm agents also could require movement of producing organisms towards target organisms, as may be accomplished in part via the replication, here cell division, of the producing organisms. In both cases, phages or antibiotic-producing organisms, at least three general mechanisms appear to be involved in anti-biofilm activity: entrance of the producing organism (or infectious agent) into the biofilm (*i.e.*, into EPS, e.g., as mediated by a phage-virion “Trojan Horse”), replication of the producing organism (and/or contact between producer and target; phage Trojan Horse strategy 2), and production of the antibacterial agents (phage Trojan Horse strategy 1), with each of these mechanisms contributing to the concentration of antibacterial activity in the vicinity of target bacteria.

The second Trojan Horse scenario, it should be noted, is no different from infections or infestations of multicellular organisms: The initiation of infection, following breach of host defenses, results in pathogen or parasite replication along with progressively greater damage to the host. Ultimately this can be more damage than was associated with the initial breach. Alternatively, this can be more damage to a host population than was associated with the initial infection, given scaling of the scenario up to an epidemiological rather than individual infection perspective. In other words, infections, infestations, or epidemics, once initiated, can have notoriously negative impacts on the affected individuals or populations, with those negative impacts in part a consequence of a localized concentrating of infectious agents.

These negative outcomes often can differ substantially from the impact of environmental toxins—which may be viewed as antibiotic equivalents from the perspective of target organisms—unless there has been some means by which those toxins also have been concentrated either within, or within the vicinity of target organisms, e.g., see [101]. Of relevance, the specificity of phage interaction with their host bacteria during the adsorption process can be viewed as a means by which phage anti-bacterial or anti-biofilm activity can be concentrated both within and within the vicinity of target organisms, the latter being the case with the just considered Trojan Horse strategies (1 and 2, respectively).

#### 4.3.3. Phages as Anti-Biofilm Agents, a Summary

Altogether, phage-mediated attacks may be effective in terms of anti-biofilm activity due to a combination of phage mobility once released from parent cells (the ability of virions to diffuse to adjacent, potentially phage-sensitive target cells), the ability of phages to repeatedly generate as well as concentrate “Reinforcements” in the course of additional infections (Trojan Horse strategies to invade individual target cells as well as to invade target cell populations), and also the ability of phages to disrupt the structure of biofilms such as via bacterial lysis. As all of these functions contribute to phage replication as predators of bacteria, natural selection acting on phage populations should strongly favor the evolution of these potentially biofilm-disrupting tendencies. In addition, some phages possess an ability to disrupt biofilms structurally via their deployment of extracellularly acting EPS-disrupting depolymerase enzymes [123]. These EPS depolymerases presumably are also maintained, by natural selection, by their allowing phages to more effectively breach bacteria-produced EPS barriers to phage adsorption. This is towards acquisition of new host cells and thereby more effective phage population growth [107].

**Figure 2:**
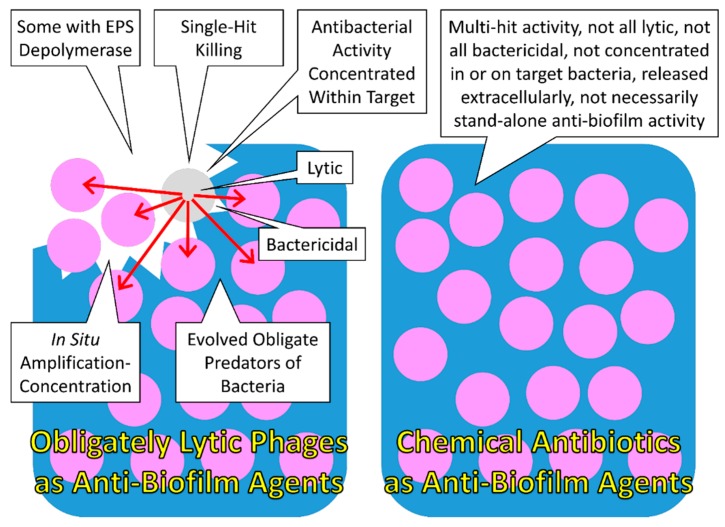
Overview of phage advantages as anti-biofilm agents in comparison to antibiotics. Bacteria are presented primarily as pink circles and extracellular polymeric substance (EPS) is represented as a blue background. Toward the upper left is a single, lysing, phage-infected bacterium, presented as a gray circle. Arrows emanating from that phage-infected bacterium represent free phages that have been produced, released, and which otherwise are diffusing towards neighboring phage-sensitive bacteria. The loss of blue background, as seen towards the upper-left of the figure, represents the action of EPS depolymerase. These depolymerase enzymes are displayed by phage virions and/or are released locally upon lysis from phage-infected bacteria. Callouts describe properties of obligately lytic phages *versus* antibiotics as anti-biofilm agents. See Table 3 for additional discussion of these properties.

These phage functions are strictly offensive rather than defensive and in principle can take place against mature biofilms, *i.e.*, “During”. The more mature a biofilm, however, then the more effort that may be required to remove a biofilm in the course of phage-mediated biocontrol or phage therapy [19]. Phages in addition display single-hit bacteria-killing kinetics. See Figure 2 and Table 3 for overview and summary of phage advantages relative to antibiotics as anti-biofilm agents.

**Table 3:** Phages as antibacterial or anti-biofilm agents relative to producers of antibiotics.

Property of Anti-biofilm Agent	As Considered in Terms of Bacteriophages	As Considered in Terms of Antibiotic Producers
Inherent predators of bacteria	Particularly for obligately lytic phages, their ability to replicate is closely associated with their ability to kill target bacteria, resulting in an antibacterial activity which is under strong selection, as evidenced by all lytic phages obligately killing target bacteria to produce new phage virions	Particularly for organisms that are not obligate predators of bacteria, their reproduction likely is *not* explicitly dependent on an ability to kill bacteria, suggesting that antibiotic production is not under as strong selection in non-predatory organisms as it is for predatory ones
Obligate predators of bacteria	The concept that losing a meal is less costly than becoming a meal, to explain differential selective pressures acting on predators *versus* prey [129], is less applicable to organisms that tend to die if they fail to succeed in exploiting a given meal, once obtained, and this tends to be the case for parasites and, by extension, for phages, *i.e.*, as host-killing parasites	For antibiotic-producing organisms, the cost associated with an antibiotic being less efficacious likely is lower than the equivalent costs to phages for less than optimal antibacterial activity because ongoing replication of antibiotic-producing organisms mostly is not absolutely dependent on inhibition of target bacteria metabolism
Concentration of antibacterial activity within the vicinity of individual target bacteria	Antibacterial action tends to be concentration dependent, as too can be antibacterial toxicity, and phages are able to concentrate their antibacterial activity not just in the vicinity of target bacteria, but within target bacteria	Concentration of antibiotics on specific targets can be more difficult to achieve for organisms that release antibiotics randomly in all directions and/or for which antibiotic release is not triggered by contact with target organisms
Concentration of antibacterial activity within spatially associated groups of target bacteria	An ability to replicate in the course of effecting antibacterial activity can allow phages to concentrate their activity spatially within phage-sensitive microcolonies or phage-sensitive cellular arrangements	Antibiotic-producing organisms also are capable of replication, including in the vicinity of target organisms, though replication by binary fission can be slower than that achievable by phages in the presence of high target-bacteria densities
Bactericidal activity	For lytic phages the death of target bacteria tends to be highly associated with antibacterial activity	Even among effective antibiotics, not all result directly in the death of target bacteria, *i.e.*, bacteriostatic agents
Lytic activity	For lytic phages the lysis of target bacteria is highly associated with antibacterial activity and can lead to sequential removal of biofilm material (e.g., leading to “Active penetration” [120])	Not all antibiotics give rise directly to the lysis of target bacteria so therefore do not necessarily directly give rise to destruction of biofilm physical structure
EPS depolymerases	Certain phages deploy enzymes that are capable of breaking down biofilm extracellular matrix	Antibiotics in and of themselves will not likely possess EPS depolymerase functions
Single-hit killing kinetics	Generally the death of sensitive bacteria follows the adsorption of only a single phage	Generally the death of sensitive bacteria requires exposure to large numbers of molecules of individual antibiotic types

## 5. Conclusions

Bacteriophages do not appear to naturally *produce* substantial quantities of antibiotic-like antibacterial agents, that is, relatively small extracellular factors that can bind to and then inhibit the metabolism of target bacteria. Bacteriophages, as extracellular agents that also can bind to and then inhibit the metabolism of target bacteria, nonetheless themselves act equivalently to such factors, though phages are more complex in their actions than traditional antibiotics. Phages as a consequence of this complexity are able to concentrate production of their antibacterial activity literally within the individual cells that they are infecting. Phage virions, also in association with this complexity, are larger in size and therefore more limited in their rates of diffusion than antibiotics or even antibacterial proteins [107]. Phages potentially can at least partially make up for the latter shortcoming, however, via their generation *in situ* of new phage virions in the course of their lysis of target cells. As a consequence, phages may be able to move relatively rapidly away from lysing cells towards adjacent but more biofilm-interior bacteria. Biofilm interior cells that have been exposed by this lysis of more exterior bacteria also may become more metabolically active and therefore better targets for antibacterial agents, including as targets for phage infection. For certain bacteriophages, production of EPS depolymerases may result in further clearance of biofilm-associated material than the lysis of target biofilm bacteria can accomplish alone.

Antibiotic-producing organisms tend to be even larger than phages, and also replicate more slowly, though to a degree may be able to make up for such deficits by individually producing or displaying a variety of antibacterial agents or mechanisms. Antibiotic-producing organisms also in many cases can display active motility plus may be able to slowly move towards target bacteria within biofilms in the course of their cell division. Nevertheless, most bacteria likely are less effective in their delivery of antibacterial agents to target bacteria than phages because that delivery tends to be extracellular and not necessarily stimulated by contact between antibacterial producers and target bacteria. Antibacterial producing organisms might be able to make up for this inefficiency, however, by crowding up against target bacteria within biofilms, thereby allowing for more direct if still extracellularly applied antibacterial delivery.

In general, the key to effective antibacterial activity likely is: (1) production of sufficiently active antibacterial agents along with (2) gaining and then maintaining intimate access to target organisms such that effective antibacterial delivery may be accomplished. The latter may be achieved through phage adsorption, penetration of antagonistic organisms into or through biofilm matrix, or instead simply a crowding of antibacterial producers up against these targets. Particularly useful may be an ability to then (3) concentrate antibacterial activity to sufficient levels following that access. For ecologically profitable biofilm eradication, (4) removal of biofilm material likely must be accomplished as well. Bacteriophages as antibacterial agents appear to be better able to meet these criteria for effective antibacterial activity, including in association with biofilms, than antibiotics acting especially in isolation from both their producing organisms and other antibacterial factors. New approaches to antibiotic delivery to the interior of biofilms and/or use of biofilm matrix-disrupting agents may be narrowing this difference. Nonetheless, from an ecological perspective it appears that phages as well as other predators of bacteria may inherently display more effective anti-biofilm activity, even when used in isolation of other factors, than especially can specific antibiotics as equivalently employed. For further consideration of the ecology of phage-biofilm interaction as well as phage use explicitly as anti-biofilm agents, see the companion to this article [19].
